# Branch retinal artery occlusion due to patent foramen ovale in a teenager

**DOI:** 10.1113/EP093440

**Published:** 2026-04-30

**Authors:** Jie Zu, Fang Fan, Jiayao Wang, Jianmin Wang, Zhiyang Jia

**Affiliations:** ^1^ Hebei General Hospital Hebei Medicine University Shijiazhuang China

**Keywords:** branch retinal artery occlusion, paradoxical embolus, patent foramen ovale, thrombolytic therapy

## Abstract

This report demonstrates the diagnostic evaluation and clinical management of a 16‐year‐old girl who developed branch retinal artery occlusion secondary to patent foramen ovale. The goal is to increase awareness of this rare presentation in adolescents among clinicians, with a summary of the important clinical features, multimodal imaging findings and therapeutic strategies that allowed for timely reperfusion and subsequent visual recovery.

## Abstract (highlights)

- **What is the main observation in this case?**
  By describing the aetiology, intervention and clinical outcome of branch retinal artery occlusion in an otherwise healthy adolescent, this report challenges the current assumption that retinal arterial occlusion occurs mostly in older individuals with atherosclerotic risk factors.
- **What is insights does it reveal?**
  The results indicate that right‐to‐left shunting via a patent foramen ovale might precipitate retinal arterial occlusion in younger patients and highlight the importance of considering paradoxical embolism in the differential diagnosis of acute visual loss in this group. Recognition of this mechanism broadens the range of possible aetiologies and helps to develop age‐specific diagnostic and therapeutic strategies.

## 1 INTRODUCTION

Branch retinal artery occlusion (BRAO) is a serious eye condition that can cause serious visual impairment. It occurs more often in middle‐aged and elderly people with dyslipidaemia and cardiovascular disease, whereas cases in adolescents are rare and differ from adult BRAO in terms of pathogenesis, precipitating factors, diagnosis, treatment and prognosis.

## 2 PARTICIPANT INFORMATION

A 16‐year‐old girl came to the ophthalmology department of Hebei General Hospital 20 h after experiencing a sudden painless loss of the inferior visual field of the right eye. She reported no history of ocular or systemic disease and denied associated symptoms such as headache, tinnitus or trauma.

## 3 FINDING

**FIGURE 1:**
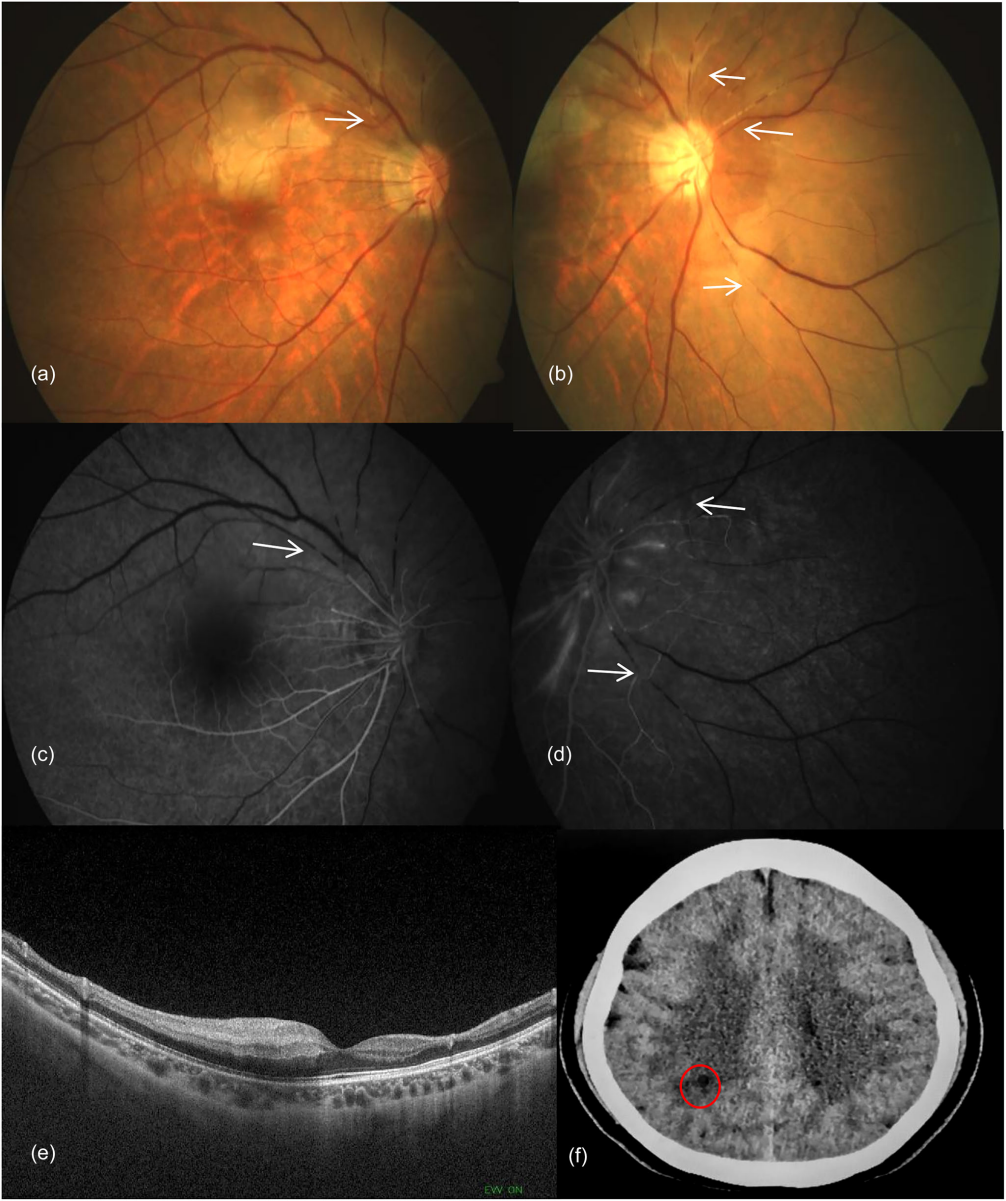
(a, b) Fundus photography shows thinning of the retinal artery; white arrows indicate segmental occlusion of the retinal artery in the right eye(OD). The nasal and superior retina appear grey–white and oedematous, and the retina above the macula is also grey–white and oedematous. (c, d) Fluorescein angiography demonstrates a filling defect in the arterial phase in the superior hemisphere and nasal region, with white arrows indicating non‐perfused vessels. (e) Optical coherence tomography shows retinal oedema and thickening above the fovea. (f) Non‐contrast cranial CT demonstrates a lacunar infarct in the right parietal lobe, with a circle marking the location of the infarction.

Best‐corrected visual acuity (BCVA) was 2/20 in the right eye (OD) and 30/20 in the left eye (OS). Examination of the anterior segment was unremarkable; however, evaluation of the fundus showed superior and nasal retinal whitening, in addition to a segmented and truncated branch retinal artery (Figure 1a, b).

Fluorescein angiography of the fundus showed filling defects in the superior and nasal branch retinal arteries, with slight fluorescein leakage in the late phase (Figure 1c, d). Optical coherence tomography showed retinal thickening and hyperreflectivity from the nerve fibre layer to the outer nuclear layer over the fovea (Figure 1e). Carotid duplex sonography was unremarkable, and transthoracic echocardiography revealed trivial mitral and tricuspid regurgitation. Non‐contrast cranial CT revealed a lacunar infarct to the right parietal lobe (Figure 1f). Laboratory evaluations, including complete blood count, coagulation profile and lipidoids, were within normal limits, and the patient denied the use of oral contraceptives. Menstrual history was regular, with the last menses 20 days previously. Although the diagnosis of BRAO was made, the aetiology of the changes was initially unclear.

## 4 TIMELINE

**Table:** 

Stage	Time	Activity
Case discovery	December 2024	A 16‐year‐old girl presented 20 h after sudden, painless loss of the inferior visual field in her right eye.
Intervention	December 2024	The patient underwent intra‐arterial thrombolysis via ultrasound‐guided femoral artery access
Diagnostic assessment	December 2024	Right heart contrast echocardiography demonstrated a grade III right‐to‐left shunt, suggesting patent foramen ovale as the likely embolic source
Follow‐up	December 2024–March 2025 December 2025	Follow‐up 12‐month telephone follow‐up

## 5 DIAGNOSTIC ASSESSMENT

**FIGURE 2:**
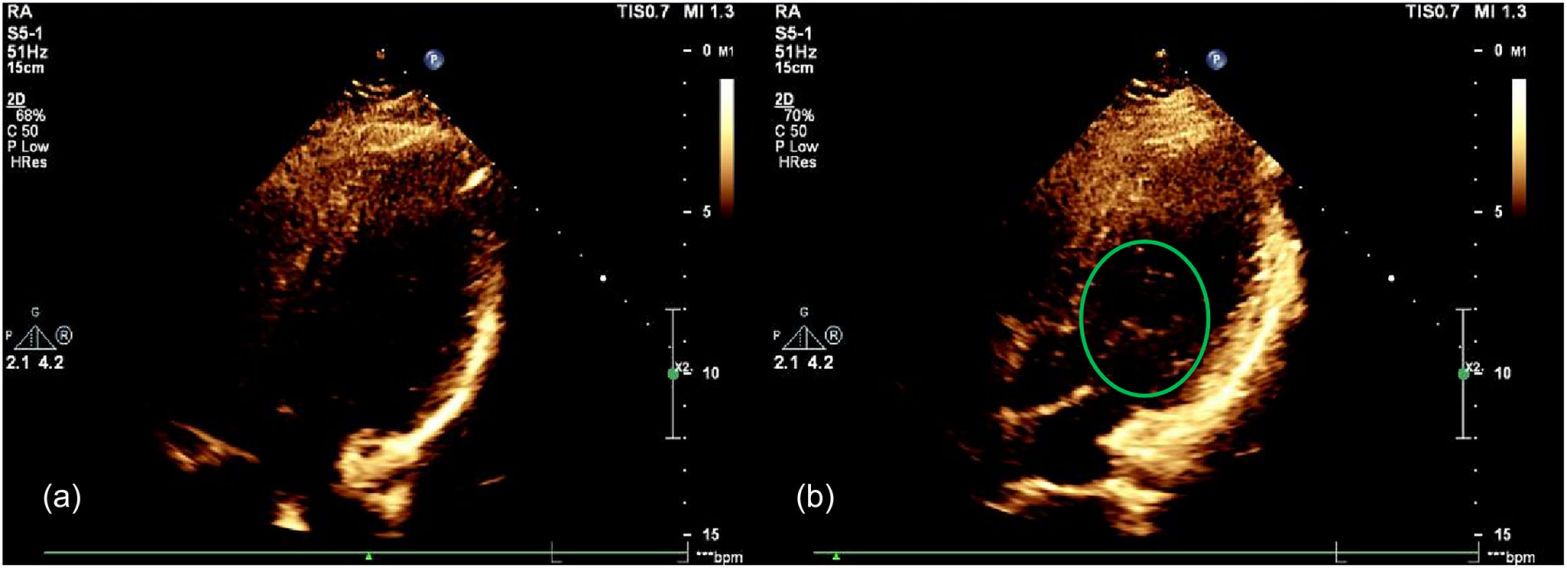
(a) Ten millilitres of agitated normal saline was injected into the median cubital vein; microbubbles were observed in the left heart at rest, with <10 bubbles captured in a single frame. (b) With increased abdominal pressure, 10 s after injection of 10 mL of agitated normal saline, microbubbles were observed in the left heart, with >30 bubbles captured in a single frame (green circle marks bubbles).

Extensive serological testing, including antinuclear antibody, anti‐2‐deoxyribonucleic acid, antiphospholipid antibodies, lupus anticoagulant, immunoglobulin levels, complement components, human leucocyte antigen‐B27 and vasculitis panels, was negative. Right heart contrast echocardiography showed a grade III right‐to‐left shunt, which implicated a patent foramen ovale (PFO) as the most likely embolic source (Figure 2a, b).

## 6 INTERVENTION

**FIGURE 3:**
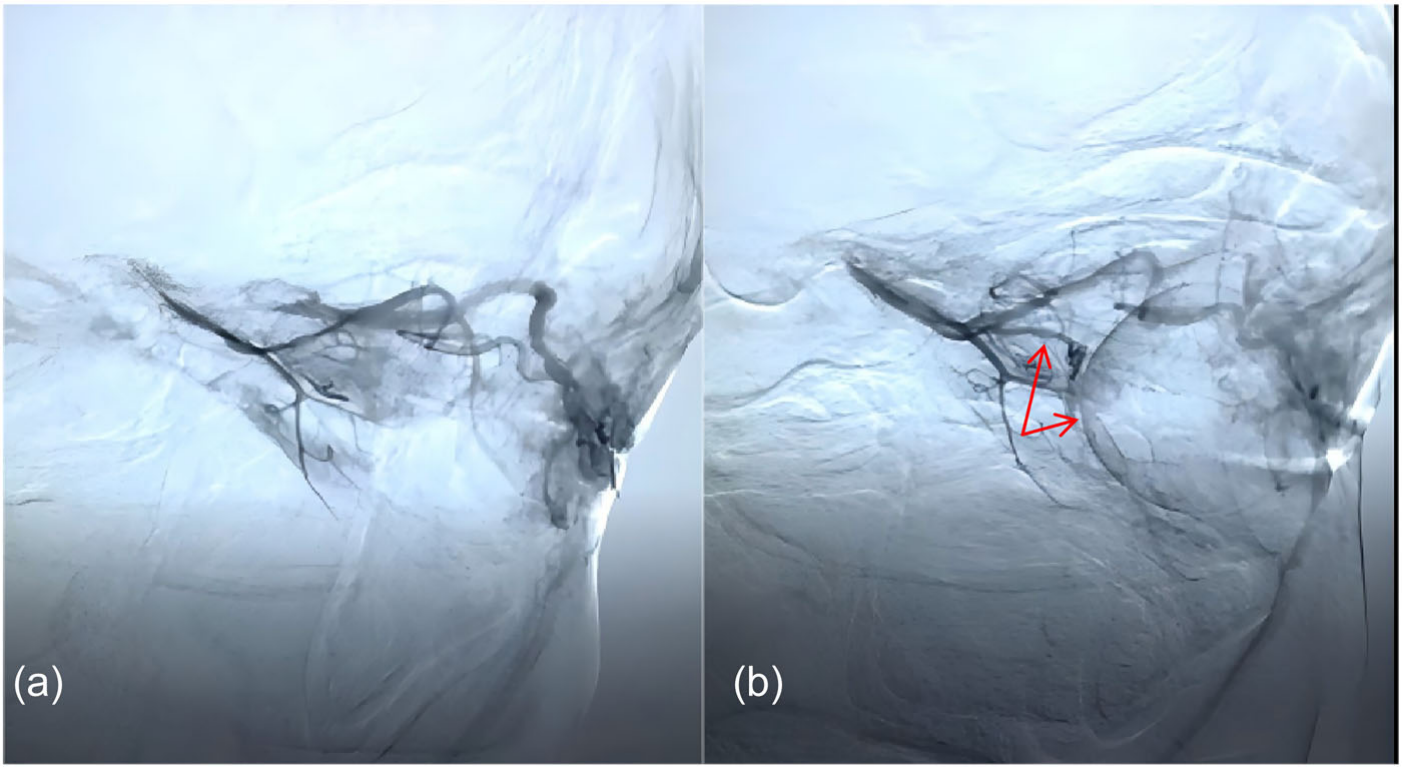
(a) Before thrombolysis, retinal arterial perfusion was markedly reduced, with only faint proximal flow detectable and poor visualization of distal branches. (b) After thrombolysis, retinal arterial perfusion improved, with clearer visualization of the arterial tree. Red arrows indicate recanalization.

Within 24 h of the onset of symptoms, the patient was treated with intra‐arterial thrombolysis using ultrasound‐guided femoral artery access following multidisciplinary evaluation and clearance. A microcatheter was advanced into the ophthalmic artery and intermittent injections of 5 mg of recombinant human prourokinase were given over the course of 15–20 min, along with 30 mg of papaverine. The previously occluded retinal arterial network was patent after the procedure (Figure 3a, b), and the patient reported immediate subjective improvement in vision. Adjunctive ocular therapy included topical antiglaucoma medication, retrobulbar vasodilator injection and systemic anticoagulation. BCVA improved to 6/20 on post‐procedure day 1. No bleeding or other complications were seen during or after the intervention.

Subcutaneous enoxaparin (1 mg/kg every 12 h) was started to prevent re‐thrombosis. Concurrently, the affected eye was given a daily retrobulbar injection of scopolamine, with intravenous vasodilator therapy to improve ocular perfusion. An extensive work‐up for hypercoagulable states, autoimmune vasculitis and structural cardiac anomalies was done in parallel, while visual recovery was monitored closely on a daily basis.

## 7 FOLLOW‐UP AND OUTCOMES

**FIGURE 4:**
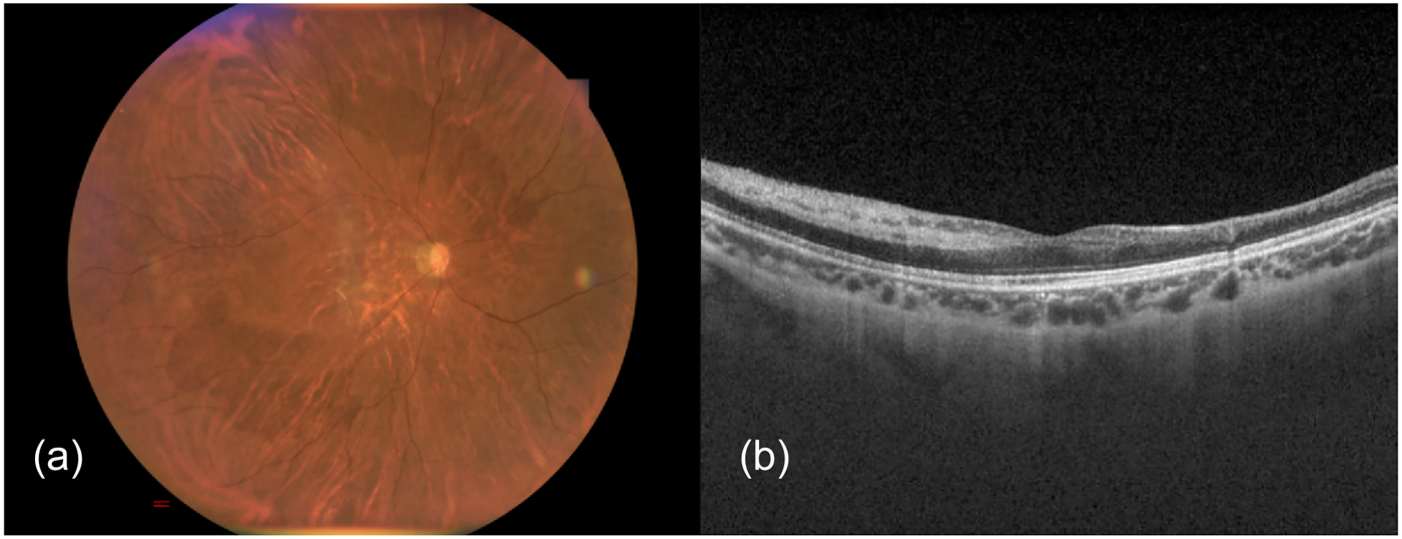
(a) Retinal oedema has decreased markedly compared with baseline, and most retinal vascular perfusion has returned to normal. (b) Macular oedema has decreased compared with baseline (1 month after onset).

Seven days after treatment, BCVA was improved to 16/20, with only a minor residual nasal visual field defect. At 1 month follow‐up, she had a percutaneous closure of the PFO without complications. Three months later, BCVA was still 16/20, and the optical coherence tomography showed resolution of the macular oedema (Figure 4a, b). At the 12‐month follow‐up by telephone, her vision was stable, and no new ischaemic events had occurred.

## 8 DISCUSSION AND LIMITATIONS

BRAO usually occurs in middle‐aged or elderly people with hypertension, dyslipidaemia or diabetes and rarely occurs in adolescents. In younger patients, the differential diagnosis should include rheumatological disorders, haematological abnormalities, hypercoagulable states and congenital cardiac defects, such as a PFO. Rare associations of BRAO in this population have also been reported, including sickle cell disease (Fadugbagbe et al., 2010), antiphospholipid syndrome (Sengupta et al., 2023) and systemic lupus erythematosus (Ish et al., 2020). In the present case, the patient's thorough negative systemic work‐up, rapid visual recovery after thrombolysis, and the presence of a concomitant cerebral lacunar infarct brought the diagnosis of a paradoxical embolus passing through a PFO to the forefront.

A PFO remains in ∼15%–35% of adults when the foramen ovale does not fuse after birth (English, 2024). Although usually asymptomatic, transient increases in right atrial pressure may open the flap valve, permitting right‐to‐left shunting and the transit of venous thrombi bypassing filtration in the lungs (Geng et al., 2017). When such thrombi reach the arterial circulation, they can obstruct the vascular beds further down the chain; smaller emboli can reach the retinal artery, precipitating BRAO (Shi et al., 2024). Iqbal et al. (2020) reported a case of BRAO associated with a PFO in a healthy 31‐year‐old male. PFO‐related emboli amount to <2% of all systemic arterial emboli^.^ (Kallel et al., 2021); however, studies over the last 55 years suggest that ≤46% of cryptogenic strokes might be related to PFO (Wieder et al., 2021). Although cerebrovascular events are the most reported in the literature, ocular embolism is rarely reported, and even rarer is BRAO. In our patient, the association of retinal and cerebral infarctions occurring at the same time, in the absence of the conventional vascular risk factors, was strongly suggestive of PFO‐mediated embolism. Echocardiography confirmed the diagnosis, and ultrasonography of the lower limbs and vessels in the neck showed no residual thrombus. Emerging evidence suggests that stagnant or turbulent flow in the PFO tunnel might favour in situ thrombus formation, an alternative embolic mechanism (Shi et al., 2024). To reduce the risk of recurrent stroke, a minimally invasive procedure (percutaneous PFO closure) with high success and low complication rates was performed. Post‐procedure, rivaroxaban was prescribed for 3 months. At the 12‐month follow‐up, the patient was back to normal activities, with no evidence of new ischaemic lesions.

The American Heart Association describes CNS infarction as death of cells in the brain, spinal cord or retina owing to ischaemia (Sacco et al., 2013). This framework makes retinal infarction pathophysiologically similar to cerebral infarction and reflects common ischaemic mechanisms. Thrombolysis is the mainstay of treatment for acute ischaemic stroke and is increasingly extrapolated to BRAO. A review of the literature suggests that ∼56% of patients will show significant improvement in vision after thrombolysis, with better outcomes when treatment is started within 6 h of the onset of symptoms (Dumitrascu et al., 2020; Lele et al., 2022). Aldrich et al. (2008) found that 66% of patients receiving intra‐arterial thrombolytic therapy within 15 h of symptom onset regained visual acuity better than 20/200. Another retrospective study showed that 53% of patients treated within 12 h experienced at least a three‐line improvement in visual acuity (Sobol et al., 2021). Some scholars have pointed out that the timing of arterial thrombolysis is unrelated to visual prognosis and might be related to the degree of retinal artery occlusion (Page et al., 2016). Notably, prospective studies are still necessary to substantiate these findings and determine optimal patient selection criteria for the endovascular treatment. Although our patient presented later than the traditional 6 h time frame, her young age, good general health and preserved retinal structure were factors in choosing off‐label intra‐arterial thrombolysis, which led to rapid reperfusion and sustained improvement in vision. These observations suggest that selected adolescents with acute BRAO might benefit from thrombolysis even outside the traditional time frame when clinical conditions are favourable. However, it is noteworthy that the decision to delay thrombolytic therapy in clinical practice remains controversial, necessitating rigorous patient selection and thorough communication with patients.

Cases of BRAO attributable to PFO in adolescents are extremely rare, and no large‐scale studies have been undertaken to date.

## 9 CONCLUSION

In adolescents with symptoms of BRAO and no apparent systemic risk factors, clinicians should be alert to the possibility of a PFO as a potential source of paradoxical thromboembolism. Early treatment with a thrombolytic might be crucial in saving visual function.

## AUTHOR CONTRIBUTIONS

Conceptualization, investigation, writing—original draft, visualization: Jie Zu. Investigation, data curation: Jiayao Wang, Fang Fan and Jianmin Wang. Writing—review & editing, project administration: Zhiyang Jia. All authors approved the final version of the manuscript and agree to be accountable for all aspects of the work in ensuring that questions related to the accuracy or integrity of any part of the work are appropriately investigated and resolved. All persons designated as authors qualify for authorship, and all those who qualify for authorship are listed.

## CONFLICT OF INTEREST

The authors declare that the research was conducted in the absence of any commercial or financial relationships that could be construed as a potential conflict of interest.
